# Long term evaluation of disease progression through the quantitative magnetic resonance imaging of symptomatic knee osteoarthritis patients: correlation with clinical symptoms and radiographic changes

**DOI:** 10.1186/ar1875

**Published:** 2005-12-30

**Authors:** Jean-Pierre Raynauld, Johanne Martel-Pelletier, Marie-Josée Berthiaume, Gilles Beaudoin, Denis Choquette, Boulos Haraoui, Hyman Tannenbaum, Joan M Meyer, John F Beary, Gary A Cline, Jean-Pierre Pelletier

**Affiliations:** 1 Osteoarthritis Research Unit, University of Montreal Hospital Centre, Notre-Dame Hospital, Department of Medicine, University of Montreal, Montreal, Quebec, Canada; 2 University of Montreal Hospital Centre, Notre-Dame Hospital, Department of Radiology, University of Montreal, Montreal, Quebec, Canada; 3 University of Montreal Hospital Centre, Notre-Dame Hospital, Department of Physics and Biomedical Engineering, University of Montreal, Montreal, Quebec, Canada; 4 University of Montreal Hospital Centre, Notre-Dame Hospital, Department of Medicine, University of Montreal, Montreal, Quebec, Canada; 5 McGill University Health Centre, Montreal General Hospital, Department of Medicine, McGill University, Montreal, Quebec, Canada; 6 Procter & Gamble Pharmaceuticals, Mason, Ohio, USA

## Abstract

The objective of this study was to further explore the cartilage volume changes in knee osteoarthritis (OA) over time using quantitative magnetic resonance imaging (qMRI). These were correlated with demographic, clinical, and radiological data to better identify the disease risk features. We selected 107 patients from a large trial (n = 1,232) evaluating the effect of a bisphosphonate on OA knees. The MRI acquisitions of the knee were done at baseline, 12, and 24 months. Cartilage volume from the global, medial, and lateral compartments was quantified. The changes were contrasted with clinical data and other MRI anatomical features. Knee OA cartilage volume losses were statistically significant compared to baseline values: -3.7 ± 3.0% for global cartilage and -5.5 ± 4.3% for the medial compartment at 12 months, and -5.7 ± 4.4% and -8.3 ± 6.5%, respectively, at 24 months. Three different populations were identified according to cartilage volume loss: fast (n = 11; -13.2%), intermediate (n = 48; -7.2%), and slow (n = 48; -2.3%) progressors. The predictors of fast progressors were the presence of severe meniscal extrusion (*p* = 0.001), severe medial tear (*p* = 0.005), medial and/or lateral bone edema (*p* = 0.03), high body mass index (*p* < 0.05, fast versus slow), weight (*p* < 0.05, fast versus slow) and age (*p* < 0.05 fast versus slow). The loss of cartilage volume was also slightly associated with less knee pain. No association was found with other Western Ontario McMaster Osteoarthritis Index (WOMAC) scores, joint space width, or urine biomarker levels. Meniscal damage and bone edema are closely associated with more cartilage volume loss. These data confirm the significant advantage of qMRI for reliably measuring knee structural changes at as early as 12 months, and for identifying risk factors associated with OA progression.

## Introduction

With the aging of the world population, osteoarthritis (OA) is becoming an increasingly common cause of disability [1,2] Diarthrodial joint damage assessment of the knee joint in particular is crucial for monitoring OA disease progression and for eventually evaluating the therapeutic effect of disease modifying osteoarthritis drugs (DMOADs) on its anatomical structure. Improvements in the standardization and interpretation of knee and hip radiographs have produced more accurate measurements of both joint space width (JSW) and the progression of joint space narrowing [3]. X-ray data from a recent study [4], however, showed that OA disease progression, especially in the knee joint, is heterogeneous; only 13.2% of the 2,483 knee OA patients followed for 24 months could be characterized as progressors (which might be of clinical significance), as defined by a changed JSW outside of the measurement error (JSW change >0.6 mm). Therefore, the use of JSW changes in knee OA studies is such that a minimum follow-up of at least 24 months and a cohort of several thousands is necessary to establish the effect of pharmacological interventions on OA disease progression.

Magnetic resonance imaging (MRI) allows for the precise visualization of joint structures such as cartilage, bone, synovium, ligaments, and menisci, as well as their pathological changes. Our group [5,6], among others [7-12], have recently developed and validated a system capable of quantifying knee cartilage volume using MRI acquisitions combined with dedicated software. Data showed [13] that rapid disease progression might have been predicted at the outset of the study based on certain clinical variables: being female, having a high body mass index (BMI), experiencing a higher level of pain and stiffness, and having reduced joint mobility. Fast disease progression was further predicted by concomitant meniscal damage, mainly in the form of tears and meniscal extrusions [14]. The simultaneously collected standardized knee radiographs displayed no correlation, however, between the changes in JSW and the concomitant loss of cartilage volume [13].

A large clinical trial assessing the effects of a bisphosphonate on knee OA structural changes was recently completed. A subset of 110 of these patients underwent MRI in addition to the clinical standardized radiograph and biomarker evaluations, as per the study protocol. In this longitudinal study, we assessed this larger cohort of OA patients and identified risk factors for greater disease progression. The cartilage volume changes contrasted with the clinical, radiological, and biomarker data. We felt it was necessary to confirm our previous results [13,14] and provide new and clinically relevant information from this larger patient cohort recruited through an OA clinical trial and, therefore, corresponding to a more stringent inclusion and exclusion criteria. Moreover, the results would also confirm the applicability of MRI cartilage volume quantification to the day-to-day reality of a clinical trial.

## Materials and methods

### Patient selection

A subset of 110 patients was selected from 1,232 patients enrolled in a large clinical trial evaluating the impact of a bisphosphonate on knee OA. This specific subset of patients was recruited from the outpatient Rheumatology Clinic at the University of Montreal Hospital Centre (CHUM), Notre-Dame Hospital, and from the Rheumatic Disease Centre of Montreal, both in Montreal, Quebec, Canada. Both male and female patients were eligible for the study if they were between 40 and 80 years old, fulfilled the American College of Rheumatology criteria for knee OA [15], and had symptomatic disease that required medical treatment in the form of acetaminophen, traditional nonsteroidal anti-inflammatory drugs (NSAIDs), or selective cyclooxygenase-2 inhibitors. Eligible patients were required to display radiological evidence of OA of the affected knee on a radiograph obtained within six months of the outset of the study. Finally, patients had to have a minimum JSW of the medial compartment of between 2 and 4 mm, at least one osteophyte, and a narrower medial compartment compared to the lateral compartment. The measurements were done from a baseline film using the standardized semi-flexed view, which was contrasted with follow-up films. No patient had sole lateral compartment disease.

Patients were excluded if they had chondrocalcinosis or an acute or chronic infection (including tuberculosis); if their OA of the knee was secondary to other conditions, including inflammation, sepsis, metabolic abnormalities, and trauma; or if they displayed any contraindication to the use of MRI. Further exclusion factors included patients' history of past or present gastrointestinal ulceration, their receipt of an intra-articular corticoid injection in the study knee within the six months prior to the outset of the study, as well as their classification as radiological grade IV on the Kellgren-Lawrence scale for the study knee or severe (class IV) functional disability. In patients in whom both knees were symptomatic, we chose the most symptomatic knee for the investigation. Patients were permitted to receive simple analgesics or NSAIDs, the regimens of which could be changed according to the preference of the rheumatologist and the clinical course of the patient. Such regimens, as well as any changes to them, were closely monitored and noted. Because of its potential to promote OA cartilage degeneration, the use of indomethacin was not permitted [16]. A centralized ethics committee approved this study, and each patient gave informed consent.

### Clinical evaluation

Patients underwent clinical evaluation at baseline and every 6 months thereafter until 24 months. They were first evaluated on the basis of the Western Ontario McMaster Osteoarthritis Index (WOMAC), a tri-dimensional self-administered questionnaire that probes pain (5 items), stiffness (2 items), and physical function (17 items) [17]. Its French-Canadian translation has been fully validated and established as reliable [18]. In addition, the patients themselves used a visual analog scale to make a global assessment of their condition (patient global assessment: 0 = very good; 100 = very bad) and to rate the pain they were experiencing that day (patient pain score: 0 = no pain; 100 = most severe pain). Finally, the SF-36, a generic quality of life instrument, was administered to the patients at each visit [19]. A washout of medications was done prior to the clinical evaluation; NSAIDs were discontinued at least 48 hours prior to the investigation and acetaminophen, 24 hours. The clinical evaluators were blinded to the results of previous radiological or MRI data.

### Knee X-rays

The JSW of the target knees was evaluated at baseline and at 12 and 24 months of follow-up, at the narrowest point in the medial tibio-femoral compartment according to the published protocol [20]. This protocol allows for the standardization of radiographs by positioning the knee in a semi-flexed position under fluoroscopic guidance and by fixing a metal sphere to the fibula head to correct the effects of the radiographic magnification. The films were digitized using a Lumiscan 200 laser film digitizer (Lumisys Inc., Sunnyvale, CA, USA), prior to which all films were bar-coded to ensure that, on digitization, the computer database would link patient/visit data to the JSW measurement obtained from each radiograph. Each of the radiographs measured the minimum JSW in the medial compartment using the automated computerized method of measurement [21]. In the rare occurrence that the radiographic quality of the film prevented the implementation of automatic JSW measurement software, manual intervention was required. In such cases, manual intervention ensured reliable JSW measurement by aiding the algorithm to trace the articular contour [22]. The variation coefficient for JSW measurement for the original reliability study was 1% for repeat radiographs (test/retest) of the knee in the semi-flexed position [20]. The reproducibility of the method was also reassessed recently by Buckland-Wright and colleagues [23]; data showed that 45% of the examinations achieved high quality, that is, JSW difference between repeat films <0.1 mm, and 92% achieved excellent to good quality with a difference between repeat films <0.3 mm.

### Knee MRI

High-resolution, three-dimensional MRI was obtained for each OA patient at baseline and at 12 and 24 months using the commercially available Magneton Vision 1.5 Tesla machine with a dedicated knee coil (Siemens, Erlangen, Germany), as previously described [5,13] These exams are optimized three-dimensional fast inflow with steady state precession (FISP) acquisitions with fat suppression. The positioning protocol, image processing, and registration were as formerly described [5,13] This registration procedure previously demonstrated excellent intra- and inter-reader correlations [5]. The OA patient repositioning, intra-reader performance precision and root mean square coefficient of variation (RMS CV%) using registration of the paired images for the repeated measures were 2.2% for the global cartilage volume, 1.2% for the medial compartment, and 2.6% for the lateral compartment [13]. These findings were very similar to results published by other research groups [9,24] The MRI acquisitions (baseline versus follow-up acquisition) were read paired, but blinded to the order of the acquisitions: 12 and 24 months.

The change in cartilage volume over time was calculated compared to baseline in absolute values (mm^3^) and in percentage values for the entire knee (global) and for each of the knee compartments (medial compartment: summation of the medial femoral condyle and tibial plateau volume; lateral compartment: summation of lateral femoral condyle and tibial plateau volume, femoral compartment: summation of medial and lateral femoral condyle and tibial compartment: summation of medial and lateral tibial plateau), respectively.

### Meniscal damage and bone edema

The meniscal and bone evaluation was performed using the same sequences as for the cartilage assessment, as previously discussed [14]. Regardless, the FISP sequence enabled us to visualize the meniscal tissue and bone lesions with enough clarity to adequately and reliably perform the semi-quantitative scoring system [14]. A semi-quantitative lesion assessment of meniscal damage and bone edema was performed. Knee menisci and bone lesions were evaluated by an experienced radiologist (MJB) who was blinded to the time sequences and cartilage volumes, while the cartilage volume assessment was performed separately by two different readers who were blinded to the radiologist's grading.

Our scoring system for meniscal damage referred to the accepted MRI nomenclature for meniscal anatomy, which is in accordance with arthroscopic literature [25,26]. The proportion of the menisci affected by degeneration, tear, or extrusion was scored separately using the following semi-quantitative scale [14]: 0 = no damage; 1 = 1 out of 3 meniscal areas involved (anterior, middle, posterior horns); 2 = 2 out of 3 involved; 3 = all 3 areas involved. The extent of meniscal extrusion on the medial or lateral edges of the femoral tibial joint space, not including the osteophytes, was evaluated for the anterior, middle, and posterior horns of the menisci in which 0 = no extrusion, 1 = partial meniscal extrusion, and 2 = complete meniscal extrusion with no contact with the joint space.

For bone edema, the intensity and extent of the lesion was assessed in the medial and lateral tibio-femoral compartments with the following semi-quantitative scale: 0 = absence of edema; 1 = mild to moderate edema, meaning a small or medium-sized lesion; and 2 = severe edema, meaning a large one. The results are presented by either presence or absence of any edema (grade 1 or 2) and presence or absence of one severe edema lesion (grade 2 only), regardless of the presence of additional smaller lesions.

Reliability of our scoring system for meniscal and bone changes was excellent. The intra- and inter-reader correlation coefficient ranged from 0.86 to 0.96 for the meniscal tear, 0.85 to 0.92 for the meniscal extrusion and 0.88 to 0.93 for the bone marrow edema. Kappa statistics ranged from 0.79 to 0.89 for the meniscal changes and 0.78 to 0.87 for the bone marrow edema (data not shown).

### Biomarkers

Level of urinary C-terminal cross-linking telopeptide of collagen type II (U-CTX-II), a biological marker of collagen type II degradation, was measured by a specific ELISA [27]. Early morning fasting second void urine samples were collected at baseline, month 6, 12 and 24 (exit). The samples were taken between 6 hours and 21 hours and not necessarily collected at the same time of the day for each patient. Inter- and intra-variability was lower than 10% for the assay.

### Statistical analysis

All of the data (clinical, radiological, and laboratory) were systematically entered into a computerized database using a blinded double-entry procedure, after which descriptive statistics for patient characteristics were tabulated. The primary variable of interest for this publication was the change in cartilage volume over time for the entire knee (global) and for each of the knee compartments (medial or lateral), respectively. The cartilage volume losses are presented as percentage losses compared to baseline (mean ± standard deviation) and statistical relevance assessed by a one-sample *t *test.

A K-means cluster analysis, a non-parametrical statistical method, was used to identify subgroups of disease progression based on cartilage volume loss at 24 months. These subgroups were further analyzed to contrast their baseline demographic, clinical, radiological, and biomarker features, and presented as mean ± standard deviation. Non-parametric Wilcoxon one-sample tests, one-sample and two-sample Student *t *tests, chi-squares, or the McNear exact test were performed to assess statistical significance. Multivariate linear analyses were used to assess predictors of cartilage volume loss independently from potential confounders like age, gender and BMI. Further analyses were done by dividing the cohort by quartiles of cartilage volume loss in which the first quartile demonstrated greater cartilage volume loss. Finally, the relationship between cartilage volume loss and the change in JSW was explored at 24 months using the Spearman correlation test. All statistical analyses were done using Statistica, version 6 packages (StatSoft, Tulsa, OK, USA). All tests were two-sided, and a *p *value = 0.05 was considered statistically significant. Analyses were not corrected for multiple comparisons.

## Results

### Patient characteristics

A subgroup of 110 patients was assessed with quantitative MRI (qMRI); three patients were lost to follow-up early in the study. At baseline, the cohort was largely in line with the demographic and disease characteristics of a general OA population: the mean age was 62.4 ± 7.5 years, 64% of subjects were female, subjects had an average BMI of 30.6 ± 4.3 kg/m^2^, the duration of knee OA was 8.9 ± 7.2 years, 91.4% were taking analgesics, and 72% were using NSAIDs, and these patients were exhibiting disease activity scores in the mild to moderate range according to the WOMAC (total, 38.9 ± 22.9), the Patient Global (visual analog scale, 48.2 ± 5.0), the SF-36 (38.1 ± 9.5), and the Kellgren-Lawrence score (grade 2: 53% of the patients; grade 3: 47%). The mean JSW measure at baseline was 2.88 ± 0.64 mm. These baseline characteristics of the 110 subjects were very similar according to age, gender, BMI and baseline WOMAC pain, stiffness and function values to the 1,232 subjects enrolled in the large clinical trial. The patients represent all those enrolled in Montreal for the bisphosphonate study and all had MRI. There was no effect of any bisphosphonate treatment group on the rate of knee OA progression as measured by either cartilage volume per MRI or the JSW loss at two years (data not shown). We felt, therefore, that all the patients may be considered as a unique group and analyzed as such.

### Cartilage volume changes over time

At 12 months (Table 1), there was already a statistically significant loss of cartilage volume in the global (-3.7 ± 3.0%), medial (-5.5 ± 4.3%), and lateral compartments (-2.1 ± 2.9%) compared to baseline (*p* < 0.0001, one-sample *t *test). At 24 months, further cartilage volume loss was evident in the global (-5.7 ± 4.4%), medial (-8.3 ± 6.5%), and lateral compartments (-3.5 ± 3.8%), which were all statistically significant when compared to baseline (*p *< 0.0001). The lower rate seen for the lateral compartment may be explained by the selection of this population according to the inclusion/exclusion criteria; patients with isolated lateral compartment knee OA as defined with standing knee X-rays were excluded. The results of the femoral and tibial compartments demonstrated similar and statistically significant results and are presented in Table 1.

**Figure 1:**
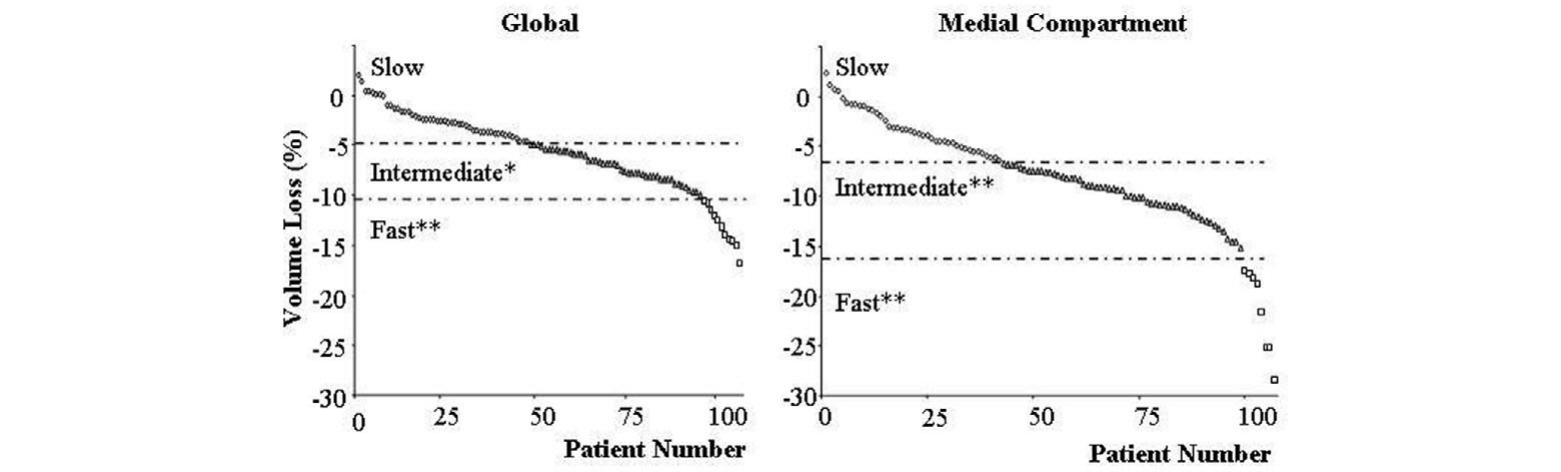
Changes in osteoarthritis cartilage volume percentage of loss from baseline after 24 months for each patient for the global knee and medial compartments of the three subgroups identified in the cluster analysis: slow (n = 48), intermediate (n = 48), and fast (n = 11) progressors. The global (and medial) volume loss at all the different time points were -2.3 ± 0.4% (-3.2 ± 0.6%) for the slow progressors, -7.2 ± 0.6% (-9.9 ± 0.1%) for the intermediate progressors, and -13.2 ± 0.4% (-21.5 ± 0.1%) for the fast progressors; the intermediate and fast progressor subgroups were found to be statistically significant when compared to baseline (*t *test). *p < 0. 001; **p < 0.0001.

With the use of cluster analysis, we identified three different subgroups of disease progression (Figure 1) at 24 months according to the global volume loss. A subgroup of 11 patients clearly demonstrated a faster progression of global cartilage volume loss (-13.2 ± 0.4%, *p *< 0.0001, *t *test compared to baseline) compared to 48 patients with an intermediate rate of cartilage loss (-7.2 ± 0.6%, p < 0.001, *t *test compared to baseline), and 48 patients with a slow loss rate (-2.3 ± 0.4%, *p *not significant). These three groups now defined as slow, intermediate or fast are labeled as such throughout the text. Among the three groups, a greater relative cartilage volume loss was observed in the medial compartment of the same patients identified as fast and intermediate groups at 24 months with -21.5 ± 0.1% and -9.9 ± 0.1% loss, respectively (*p *< 0.0001). In contrast, very little progression (-3.2 ± 0.6%) was found in the medial compartment of the slow progressors.

### Characteristics of the slow, intermediate, and fast progressors

Table 2 shows the baseline patient characteristics of the three subgroups defined by their global compartment cartilage volume loss; Table 3 shows the clinical data; and Table 4 shows the meniscal and bone change data. The data show statistical differences between some baseline variables of patients with fast and slow progression (Table 2), including age, higher weight and BMI. No significant difference was seen between the groups in terms of the initial joint space width or cartilage volume. The difference between the subgroups using the WOMAC (Table 3) global and clinical variables, including pain, stiffness, and function, did not reach statistical significance either, although a possible trend toward a worse baseline condition for the fast group was shown.

For the meniscal changes, in absolute numbers, 85 patients had a meniscal medial tear and/or extrusion, 55 had a lateral tear and/or extrusion, with some patients having both menisci compartments damaged. Only six patients had an intact meniscus. The severe medial meniscal extrusion and the severe medial tear (Table 4) at baseline were strongly associated with the faster disease progression group (*p* < 0.0001; ANOVA for the three groups). This was present in 73% of the fast compared to 19% of the slow progressors for the extrusion, and in 72% and 23%, respectively, for the tear (*p *< 0.0001, Chi-squared test).

The presence of bone edema (Table 4) in the tibio-femoral at the medial and/or lateral compartment also appeared to be associated with disease progression, as it was present in 90% of the fast subgroup and in 54% of the slow progressors (*p *< 0.05, Chi-squared fast versus slow; and *p *= 0.03, ANOVA for the three groups).

No association of the presence of bone edema with clinical symptoms was found (data not shown). Analyses on the other compartments, medial, lateral, femoral, and tibial were also performed and did not provide new information (data not shown).

### Comparison of changes in cartilage volume and JSW versus clinical parameters over time

The evaluation of the clinical course of all 107 OA patients revealed no significant correlation between the changes in cartilage volume and the changes in clinical variables such as the patients' and physicians' global assessments, the pain, stiffness, and function dimensions of the WOMAC, and the physical components of the SF-36. Values of r < 0.2 and *p *> 0.25 (Spearman correlation coefficient) were found for all the variables compared to cartilage volume loss (data not shown). For the JSW changes at 24 months, some weak correlations were demonstrated for changes at 24 months with WOMAC pain score and function score changes (both with r = 0.28, *p *= 0.06).

### Multilinear regression analysis

As the medial compartment is the most closely related to the radiological changes, the results presented subsequently focus on this compartment. Multilinear regression analysis was used to investigate the association of medial cartilage volume loss at 24 months using baseline demographics, with clinical and imaging data as predictors controlling at the same time potential confounders such as age, gender, and BMI (Table 5 with all the variables and Table 6 using a forward stepwise method). The most statistically significant independent predictor of medial compartment cartilage volume loss was the severe meniscal extrusion for both models. The severe medial tear, which was associated with the fast progressors (Table 4) was not associated with cartilage volume loss in our multivariate models (Tables 5 and 6). The strong colinearity between meniscal tear and extrusion is a likely explanation for this finding.

Bone edema also showed an independent association with the cartilage loss, which was statistically significant using the stepwise method. The clinical variables, the SF-36 physical components, and the WOMAC total score also demonstrated some independent predictive values. Surprisingly, baseline medial cartilage volume was also independently associated with medial cartilage loss at 24 months. Additional multivariate analyses comparing changes in the clinical variables at 24 months and the medial compartment cartilage loss were also done (Table 7). These demonstrated an independent association between medial cartilage volume loss and simultaneous pain change at 24 months (beta coefficient -0.45, p = 0.03) and SF-36 physical components (beta coefficient 0.22, *p *= 0.04). No additional association with the change of clinical parameters was found.

### Medial compartment cartilage volume versus joint space width

**Figure 2:**
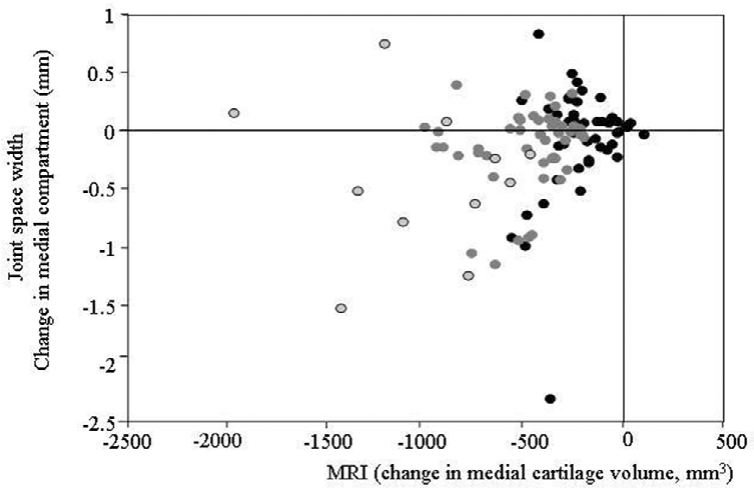
Scatter plot contrasting the changes in the medial compartment cartilage volume versus minimum joint space width measured by standardized radiograph at 24 months for 107 OA patients. Subgroups of slow (black), intermediate (dark gray), and fast (light gray) progressors are identified. No correlation between the cartilage volume and the minimum joint space width was found.

Comparison of the medial compartment cartilage global volume with the medial minimal JSW obtained by radiographs at baseline revealed some correlation between the two measurements (r = 0.26, *p *< 0.001; data not shown). At 24 months, the changes in the cartilage volume in the medial compartments (Figure 2) revealed striking differences in the progression of these parameters compared to those in the JSW. As demonstrated by MRI, while 103 patients out of 107 demonstrated a loss of cartilage greater than zero over 24 months, only 60 patients of the group showed a decrease greater than zero in the JSW. Importantly, at 24 months, only one patient demonstrated disease progression (loss greater than zero in JSW) according to the X-rays exhibiting cartilage volume increase in the MRI analysis. Caution is advised with such data interpretation, however, as values that were slightly less than zero may be within the measurement error of both techniques.

There was no apparent correlation between the cartilage volume loss changes (either by using absolute or percentage values) and the JSW changes at 24 months (global cartilage volume, r = 0.11; medial compartment cartilage volume, r = 0.19; Spearman correlation coefficient), despite further characterization of the cohort into quartiles vis-à-vis the MRI data. However, the first quartile of cartilage loss represents a group in which JSW changes were also significant (*p *< 0.001, one sample *t *test; Table 4). The addition of the presence of severe meniscal extrusion at baseline as a covariate in multilinear modeling did not change this weak relationship (r = 0.12), suggesting that the discordance is not explained by such a lesion. The data show the great variability of radiological measurement, which may explain its lack of correlation with qMRI assessments.

### Correlation between meniscal extrusion and JSW at baseline and over time

The presence of severe meniscal extrusion (score = 2), found in 38 patients at baseline, was associated with a smaller baseline JSW (2.45 ± 0.53 mm) compared to a greater JSW (3.09 ± 0.53 mm) in the absence of such an extrusion (*p* < 0.001, two-sample *t *test) (Table 9). The JSW did not further decrease in this group at 12 months once the severe meniscal extrusion was present, as with patients without meniscal extrusion. At 24 months, however, a small trend was seen. If we define patients as progressors using a loss in JSW >0.6 mm, then 10 out of 38 patients (26%) with the presence of a severe meniscal extrusion were found to meet such a criterion, whereas only 4 out of 72 patients (5%) in the non-progressor group did (JSW ≤ 0.6 mm) (*p *= 0.008, McNear exact test; data not shown).

### Cartilage volume and JSW versus urinary C-terminal crosslinking telopeptide of collagen type II

The levels of U-CTX-II, a marker of cartilage degradation, were compared with the changes in JSW and with the cartilage volume changes at 12 and 24 months. No correlations were seen, either between the level of the biomarker and the loss of cartilage volume assessed by qMRI (global, medial, and lateral compartments), or between the level of the biomarker and the changes in JSW. The r values for both ranged from -0.06 to 0.10; no *p *values were statistically significant (Spearman correlation test; data not shown). Moreover, the baseline level of U-CTX-II did not predict cartilage volume loss over time.

## Discussion

Very few studies have examined the quantitative changes in cartilage volume over time in a symptomatic knee OA population. Through the use of MRI in a longitudinal study of 107 subjects with symptomatic OA of the knee, we demonstrate a significant global cartilage volume loss at as early as 12 months. The changes in values are in line with those from a pilot study [13], and are also in accordance with the rate of progression published by another group that looked specifically at tibial cartilage volumes in a younger population of patients with knee OA [24]. The mean changes in cartilage volume seen at 12 and 24 months are relevant as they exceed the precision of our qMRI assessment (RMS CV%) as presented in our previous study [13].

The present study further reinforces the heterogeneity of the OA patient population that previous clinical trials have described [28,29] According to our patient cohort, fast progressors as assessed by qMRI may be associated with baseline clinical variables: older age, having an elevated BMI, severe meniscal extrusion and tear, and bone edema. Some of these predictors, which have already been identified in major epidemiological studies [13,14,28,30,31], make clinical sense. These variables may assist in identifying patients with disease that is likely to show marked progress over time and for whom the consideration of therapeutic interventions is crucial for preventing further joint damage. It is, however, unknown whether such a population is adequately representative of the entire patient population for monitoring the efficiency of new therapeutics in the form of DMOADs. As the disease could be cyclical, patients may experience rapid progression at some point in the course of their disease that may not reflect long-term progression.

Questions remain regarding patients with slow disease progression. It is unlikely that this process reflects merely the normal aging process, as these patients experienced pain and loss of function and met the American College of Rheumatology criteria for knee OA. We hypothesize that the slow progressors might constitute a subgroup of patients in a quiescent phase of the disease.

Many consider the measurement of the change in the minimal JSW of standardized knee radiographs to be the best available methodology for evaluating the anatomical progression of OA [3,32-34]. The present data, however, show that in only 13% of the cases, the changes in JSW at 24 months, as measured in full accordance with the Buckland-Wright protocol, demonstrated cartilage loss greater than the JSW measurement error (>0.6 mm). These radiological findings contrast with the qMRI data over the same period, where 77% of patients showed a significant loss of cartilage volume, that is, greater than the precision error of 2% (data not shown). Thus, these results suggest no strong relationship between these methods. The lack of correlation between these two parameters may be related to a larger relative variability in the JSW measurements. For example, for all patients, a mean loss of 597 mm^3 ^of global cartilage volume with a standard deviation of 459 mm^3 ^was detected at 24 months, a change that was smaller than the mean. In comparison, for the same cohort, a JSW loss of 0.16 mm with a standard deviation of 0.49 mm, roughly three times greater than the mean change, was detected at 24 months. Therefore, the 'effect size', defined as the mean change divided by its standard deviation, of qMRI assessments appears superior to that of JSW assessments.

In this study, we found some association between the extent of cartilage volume loss and changes in pain at 24 months (*p *= 0.03) when using a multivariate analysis approach, which was, however, not demonstrated by direct correlation. The patients' analgesic and NSAID washout period prior to the clinical evaluation likely reinforces the explanation of this phenomenon. Our findings are in accordance with Hunter and colleagues [35], who found that pain was associated with patellar but not with tibio-femoral cartilage loss, and somewhat with Wluka and colleagues [36], who demonstrated a weak association between the worsening of OA symptoms (knee pain and stiffness) and increased tibial cartilage loss. The relative paucity of association between symptoms and cartilage loss found in our study is perhaps unsurprising, as pain does not originate from the cartilage itself; rather, it likely originates from the surrounding bones, menisci, capsule, and ligaments. This weak association with OA symptoms may also be related to the limited number of articular features assessed by our MRI scoring system. Yet, since our cohort was relatively small, the statistical significance of our findings demonstrated only by a multivariate approach could be due to insufficient statistical power. Taken together, these data suggest that knee OA structural progression may be distinct from symptomatic changes.

We found an important relationship between cartilage volume loss and the surrounding knee tissue damage as assessed by MRI. For instance, the presence of meniscal damage, especially meniscal extrusion, was strongly associated with cartilage volume loss. In fact, 101 of our patients had at least some meniscal damage, either medial or lateral. We tried to analyze patients without meniscal damage to look at other predictors but, unfortunately, the subcohort of six patients was not large enough to yield other conclusions. The progression rate of patients without meniscal damage was -3.0% for global cartilage and -2.7% for the medial compartment. Five of these patients were classified as slow progressors and one as intermediate (data not shown).

This high level of meniscal damage (78%) may appear unusual. Our patients were selected based on inclusion/exclusion criteria for which the presence of radiological knee OA on the medial compartment plus the presence of JSW between 2 and 4 mm was necessary. It is probable that these patients may represent a more advanced disease state and that a meniscal lesion, a structure that greatly influences the JSW assessment, could almost be a prerequisite to obtain a JSW of 2 to 4 mm on standing X-rays. To further reinforce this hypothesis, our previous study on 32 subjects [13] showed a similar proportion of patients with knee meniscal damage (75%). Interestingly, inclusion in this cohort required the same JSW criteria with respect to the medial compartment. Our findings are in agreement with previous studies that reported that a significant percentage of patients with symptomatic knee OA had meniscal damage when assessed by MRI [37-39].

Cicuttini and colleagues [30] suggested that an accelerated loss of cartilage over time was evident in patients who underwent partial meniscectomy. These results suggest that the key role of the meniscal apparatus is protecting cartilage, especially in elderly subjects with obesity or joint instability. Biswal and colleagues [40] also recently studied risk factors associated with progressive cartilage loss in the knee using MRI in 43 patients. Patients were evaluated at baseline and after an average 1.8 year follow-up. This study demonstrated that meniscal and anterior cruciate ligament tears were associated with more rapid cartilage loss.

Our study also demonstrated the association between cartilage volume loss and the presence of bone edema. Felson and colleagues (([41] have already demonstrated the influence of structural changes in assessing knee OA. This group also demonstrated that bone edema as assessed by MRI was strongly associated with pain in knee OA, which was not clearly found in our study (data not shown). A possible explanation for this discrepancy may be the fact that the patient cohort recruited for our large clinical trial was less symptomatic than patients who had potentially more severe knee pain.

The lack of correlation between the U-CTX-II levels and the cartilage volume loss found in the present study contradicts other studies [4,27,42,43] The large variability in this marker, including its diurnal variation, in our cohort may explain the lack of association; nonetheless, it is still not certain whether such a marker may be more useful in larger patient cohorts followed for a longer period.

This study, like any other, has its limitations. Our cohort is representative of the average patient population with typical knee OA that is seen at a rheumatology clinic. This study is of notable confirmatory value in terms of the results obtained from a smaller number of patients [13]. However, as no therapeutic intervention to decelerate or halt disease progression has been assessed with qMRI, the extent to which such effective intervention could translate to the patients' clinical features remains unclear. As we are unaware whether all OA patients experience fast disease progression at the same time (for example, another group may have progression in year 3 while the previous progressors remain dormant), it is unclear which subpopulation could benefit the most from DMOADs. Hypothetically, favoring the treatment of patients with fast disease progression, as they likely have the poorest prognoses and the greatest need for surgical intervention, seems logical.

One may also question the potential partiality of the non-blinding of the cartilage when meniscal or bone assessment was done. However, as the radiologist evaluation was performed completely separately from the assessment of cartilage volume, it is unlikely that the grading of meniscal damage was biased by the concomitant visualization of the cartilage. We also acknowledge that the FISP sequence may not be the optimal MR sequence for identifying all the meniscal and bone lesions. It has sufficient contrast, however, to identify significant lesions, especially edema, as demonstrated in this previous work, and this acquisition has the unique advantage of being able to assess simultaneously the cartilage, menisci and bone.

## Conclusion

Our study confirms the feasibility of the long-term longitudinal follow-up of cartilage volume changes over time in a large cohort of patients with OA. Significant knee cartilage volume loss was detected as early as 12 months in this study and as early as six months in our previous study [13], thus implying that this imaging approach is much more sensitive to change than standardized radiographs. Clinical variables and non-cartilage structural joint damage may be critical for the identification of subgroups at risk of faster disease progression, a process that would facilitate patient selection for DMOAD trials. Preliminary findings assert that cartilage loss over time translates better into knee pain than other symptomatic dimensions. Cartilage degradation as measured by qMRI should reduce both the requisite number of patients in clinical trials that evaluate DMOADs and the length and overall cost of such trials, thereby improving patient retention.

## Abbreviations

BMI = body mass index; DMOAD = disease modifying osteoarthritis drug; JSW = joint space width; MRI = magnetic resonance imaging; NSAID = nonsteroidal anti-inflammatory drug; OA = osteoarthritis; qMRI = quantitative MRI; U-CTX-II = urinary C-terminal crosslinking telopeptide of collagen type II; WOMAC = Western Ontario McMaster Osteoarthritis Index.

## Competing interests

J-P Raynauld, J Martel-Pelletier, MJ Berthiaume, G Beaudoin, D Choquette, B Haraoui, H Tannenbaum and J-P Pelletier received financial support from Procter & Gamble Pharmaceuticals. J Martel-Pelletier and J-P Pelletier are consultants for Procter & Gamble Pharmaceuticals. JM Meyer, JF Beary and GA Cline are employees of Procter & Gamble Pharmaceuticals.

## Authors' contributions

JPR, JMP, MJB, GB, JMM, JFB, GAC, and JPP contributed to the study design. DC, BH, HT, and JMP were responsible for the acquisition of data. JPR, JMP, JMM, JFB, GAC, and JPP analyzed and interpreted the data. JPR, JMP and JPP prepared the manuscript. JPR and GAC performed the statistical analysis. All authors read and approved the final manuscript.

## Figures and Tables

**Table 1:** Change of cartilage volume in absolute and percentage values at 12 and 24 months of follow-up

	12 Months^a^		24 Months^a^	
	Absolute (mm^3^)	(%)	Absolute (mm^3^)	(%)

Global cartilage	-376 ± 311	-3.7 ± 3.0	-597 ± 459	-5.7 ± 4.4
Compartments				
Medial	-256 ± 211	-5.5 ± 4.3	-405 ± 320	-8.3 ± 6.5
Lateral	-119 ± 160	-2.1 ± 2.9	-191 ± 208	-3.5 ± 3.8
Femoral	-227 ± 239	-3.1 ± 3.2	-369 ± 319	-5.0 ± 4.3
Tibial	-148 ± 121	-4.9 ± 4.0	-227 ± 175	-7.6 ± 5.8
Medial				
Femoral	-126 ± 134	-5.8 ± 6.2	-201 ± 178	-9.1 ± 7.5
Tibial	-80 ± 81	-6.3 ± 5.6	-125 ± 117	-9.3 ± 7.5

Values are mean ± standard deviation. ^a^All *p *values for the 12 and 24 month follow-up <0.0001, one-sample *t *test using absolute values

**Table 2:** Characteristics of osteoarthritis patients at baseline

	Slow	Intermediate	Fast	*P *value^a^
Number	48	48	11	
Age	60.9 ± 7.5	63.0 ± 7.7	66.0 ± 5.0^b^	0.08
Female (%)	68%	64%	45%	0.35
Weight (kg)	78.8 ± 14.0	83.0 ± 14.7	89.7 ± 13.5^b^	0.06
Body mass index	29.6 ± 4.3	31.0 ± 4.3	32.6 ± 2.7^b^	0.06
Joint space width (mm)	2.98 ± 0.68	2.81 ± 0.58	2.74 ± 0.58	0.31
Global cartilage volume (mm^3^)	10,013 ± 2,512	10,425 ± 2,882	11,248 ± 2,557	0.37
Medial cartilage volume (mm^3^)	4,682 ± 1,155	4,936 ± 1,434	5,245 ± 1,165	0.36

Values are mean ± standard deviation. ^a^*p *values from ANOVA. ^b^*p *< 0.05, two-sample *t *test, fast versus slow.

**Table 3:** Clinical characteristics of osteoarthritis patients at baseline

	Slow	Intermediate	Fast	*P *value^a^
Pain	36.3 ± 22.8	34.5 ± 23.4	40.3 ± 19.3	0.74
Stiffness	43.4 ± 26.8	43.3 ± 27.8	47.5 ± 27.3	0.89
Function	36.5 ± 22.8	38.7 ± 25.7	49.3 ± 20.2	0.28
Total WOMAC	37.1 ± 22.2	38.3 ± 24.4	49.1 ± 17.6	0.29
Patient global	54.1 ± 27.9	51.6 ± 27.6	57.4 ± 18.1	0.78
SF-36 physical function	37.9 ± 9.5	39.3 ± 9.6	35.9 ± 9.1	0.39

Values are mean ± standard deviation. Patient global score 0–100, 100 = worst. Except for SF-36, 100 = best state. ^a^*p *values from ANOVA. WOMAC, Western Ontario McMaster Osteoarthritis Index.

**Table 4:** Meniscal and bone changes in osteoarthritis patients at baseline

	Slow	Intermediate	Fast	*P *value^a^
Meniscus				
Severe medial extrusion	19% (9)^b^	42% (20)	73% (8)^c^	0.001
Medial tear	73% (35)	83% (40)	90% (10)^c^	0.28
Severe medial tear	23% (11)	38% (18)	73% (8)	0.005
Lateral tear	50% (24)	54% (26)	45% (5)	0.84
Severe lateral tear	8% (4)	13% (6)	9% (1)	0.80
Bone				
Medial edema	52% (25)	60% (29)	73% (8)	0.21
Severe medial edema	4% (2)	16% (7)	18% (2)	0.20
Medial and/or lateral edema	54% (26)	73% (35)	90% (10)^d^	0.03

^a^*p *values from ANOVA. ^b^Numbers in parentheses are absolute numbers of patients. ^c^*p *< 0.0001, Chi-squared, fast versus slow. ^d^*p *< 0.05, Chi-squared, fast versus slow.

**Table 5:** Baseline parameters predicting medial compartment cartilage volume loss at 24 months: multivariate linear regression

Baseline parameter	Regression coefficient (beta)	*T *value	*P *value
Severe medial meniscal extrusion	-0.28	-2.68	0.008
Medial compartment cartilage volume	-0.36	-2.56	0.012
SF36 physical component	-0.21	-1.95	0.05
Bone edema	-0.30	-1.89	0.06
Age	-0.13	-1.35	0.18

Variables used in the model: age, gender, weight, body mass index, Western Ontario McMaster Osteoarthritis Index (WOMAC) pain, function and total score, patient global scale, SF-36 physical component, baseline medial cartilage volume, meniscal tear and extrusion, bone edema, smoking, urinary C-terminal crosslinking telopeptide of collagen type II.

**Table 6:** Baseline parameters predicting medial compartment cartilage volume loss at 24 months: stepwise forward multivariate regression

Variable entry number	Regression coefficient (beta)	*T *value	*P *value
1. Severe medial meniscal extrusion	-0.29	-3.54	<0.0001
2. Bone edema	-0.34	-2.31	0.02
3. SF-36 physical component	-0.20	-2.05	0.04
4. Total WOMAC	-0.45	-1.87	0.06

Variables used in the model: age, gender, weight, body mass index, Western Ontario McMaster Osteoarthritis Index (WOMAC) pain, function and total score, patient global scale, SF-36 physical component, baseline medial cartilage volume, meniscal tear and extrusion, bone edema, smoking, urinary C-terminal crosslinking telopeptide of collagen type II.

**Table 7:** Changes in clinical parameters associated with medial compartment cartilage volume loss at 24 months: multivariate linear regression

Baseline parameter	Regression coefficient (beta)	*T *value	*P *value
Pain	-0.45	-2.13	0.03
Stiffness	0.03	0.21	0.82
Function	-0.11	-0.24	0.81
Total WOMAC	0.52	0.88	0.37
Patient global	-0.04	0.11	0.66
SF-36 physical component	0.22	2.08	0.04

Variables also included in the model: age, gender, body mass index, Western Ontario McMaster Osteoarthritis Index (WOMAC) pain at baseline.

**Table 8:** Medial cartilage volume loss and joint space width changes at 24 months

	Cartilage volume change (mm^3^)	Joint space width change (mm)	r^a^
All	-405 ± 320	-0.16 ± 0.49	0.19
1st quartile^b^	-793 ± 342	-0.39 ± 0.52^c^	0.15
2nd quartile	-413 ± 86	-0.13 ± 0.37	0.11
3rd quartile	-277 ± 81	-0.03 ± 0.52	0.00
4th quartile	-115 ± 122	+0.02 ± 0.15	-0.21

Values are mean ± standard deviation. ^a^*p *= NS, Spearman correlation. ^b^1st quartile = greatest loss of global cartilage volume. ^c^*p *< 0.001, one sample *t *test comparing JSW change at 24 months versus its baseline value.

**Table 9:** Role of severe meniscal extrusion in joint space width and global cartilage volume changes over time

Severe extrusion^a^	Absence (n = 72)	Presence (n = 38)
Joint space width (mm)		
Baseline	3.09 ± 0.56	2.45 ± 0.53^b^
Change at 12 months	-0.05 ± 0.42	-0.07 ± 0.38^c^
Change at 24 months	-0.09 ± 0.40	-0.22 ± 0.51^c^
Global cartilage volume Loss (%)		
Change at 12 months	-3.27 ± 2.96	-4.62 ± 3.43^d^
Change at 24 months	-4.79 ± 4.00	-8.19 ± 5.05^b^

Values are mean ± standard deviation. ^a^Severe = complete meniscal extrusion. ^b^*p *= 0.001. ^c^*p *= not significant. ^d^*p *= 0.02, two samples Student *t *test comparing the absence versus presence of severe extrusion.

## References

[B1] Hart DJ, Doyle DV, Spector TD (1999). Incidence and risk factors for radiographic knee osteoarthritis in middle-aged women: the Chingford Study. Arthritis Rheum.

[B2] Lachance L, Sowers MF, Jamadar D, Hochberg M (2002). The natural history of emergent osteoarthritis of the knee in women. Osteoarthritis Cartilage.

[B3] Buckland-Wright JC (1994). Quantitative radiography of osteoarthritis. Ann Rheum Dis.

[B4] Bingham C, Cline G, Cohen G, Wenderoth D, Conaghan P, Buckland-Wright C, Beary J, Dougados M, Strand V, Meyer J (2004). Predictors of structural progression in knee osteoarthritis over 24 Months [abstract]. Arthritis Rheum.

[B5] Raynauld JP, Kauffmann C, Beaudoin G, Berthiaume MJ, de Guise JA, Bloch DA, Camacho F, Godbout B, Altman RD, Hochberg M (2003). Reliability of a quantification imaging system using magnetic resonance images to measure cartilage thickness and volume in human normal and osteoarthritic knees. Osteoarthritis Cartilage.

[B6] Kauffmann C, Gravel P, Godbout B, Gravel A, Beaudoin G, Raynauld J-P, Martel-Pelletier J, Pelletier J-P, DeGuise JA (2003). Computer-aided method for quantification of cartilage thickness and volume changes using MRI: Validation study using a synthetic model. IEEE Trans Biomed Eng.

[B7] Eckstein F, Adam C, Sittek H, Becker C, Milz S, Schulte E, Reiser M, Putz R (1997). Non-invasive determination of cartilage thickness throughout joint surfaces using magnetic resonance imaging. J Biomech.

[B8] Hohe J, Faber S, Stammberger T, Reiser M, Englmeier KH, Eckstein F (2000). A technique for 3D *in vivo* quantification of proton density and magnetization transfer coefficients of knee joint cartilage. Osteoarthritis Cartilage.

[B9] Hyhlik-Durr A, Faber S, Burgkart R, Stammberger T, Maag KP, Englmeier KH, Reiser M, Eckstein F (2000). Precision of tibial cartilage morphometry with a coronal water-excitation MR sequence. Eur Radiol.

[B10] Peterfy CG, van Dijke CF, Janzen DL, Gluer CC, Namba R, Majumdar S, Lang P, Genant HK (1994). Quantification of articular cartilage in the knee with pulsed saturation transfer subtraction and fat-suppressed MR imaging: optimization and validation. Radiology.

[B11] Cicuttini F, Forbes A, Asbeutah A, Morris K, Stuckey S (2000). Comparison and reproducibility of fast and conventional spoiled gradient-echo magnetic resonance sequences in the determination of knee cartilage volume. J Orthop Res.

[B12] Frank LR, Wong EC, Luh WM, Ahn JM, Resnick D (1999). Articular cartilage in the knee: mapping of the physiologic parameters at MR imaging with a local gradient coil – preliminary results. Radiology.

[B13] Raynauld JP, Martel-Pelletier J, Berthiaume MJ, Labonté F, Beaudoin G, de Guise JA, Bloch DA, Choquette D, Haraoui B, Altman RD (2004). Quantitative magnetic resonance imaging evaluation of knee osteoarthritis progression over two years and correlation with clinical symptoms and radiologic changes. Arthritis Rheum.

[B14] Berthiaume MJ, Raynauld JP, Martel-Pelletier J, Labonté F, Beaudoin G, Bloch DA, Choquette D, Haraoui B, Altman RD, Hochberg M (2005). Meniscal tear and extrusion are strongly associated with the progresion of knee osteoarthritis as assessed by quantitative magnetic resonance imaging. Ann Rheum Dis.

[B15] Altman RD, Asch E, Bloch DA, Bole G, Borenstein D, Brandt KD, Christy W, Cooke TD, Greenwald R, Hochberg M (1986). Development of criteria for the classification and reporting of osteoarthritis. Classification of osteoarthritis of the knee. Arthritis Rheum.

[B16] Huskisson EC, Berry H, Gishen P, Jubb RW, Whitehead J (1995). Effects of antiinflammatory drugs on the progression of osteoarthritis of the knee. LINK Study Group. Longitudinal investigation of nonsteroidal antiinflammatory drugs in knee osteoarthritis. J Rheumatol.

[B17] Bellamy N, Buchanan WW, Goldsmith CH, Campbell J, Stitt LW (1988). Validation study of WOMAC: a health status instrument for measuring clinically important patient relevant outcomes to antirheumatic drug therapy in patients with osteoarthritis of the hip or knee. J Rheumatol.

[B18] Raynauld JP, Bellamy N, Choquette D (1994). A French-version of the WOMAC questionnaire. Arthritis Rheum.

[B19] Ware JE, Sherbourne CD (1992). The MOS 36-item short-form health survey (SF-36). I. Conceptual framework and item selection. Med Care.

[B20] Buckland-Wright JC, Macfarlane DG, Williams SA, Ward RJ (1995). Accuracy and precision of joint space width measurements in standard and macroradiographs of osteoarthritic knees. Ann Rheum Dis.

[B21] Buckland-Wright JC, Macfarlane DG, Jasani MK, Lynch JA (1994). Quantitative microfocal radiographic assessment of osteoarthritis of the knee from weight bearing tunnel and semiflexed standing views. J Rheumatol.

[B22] Lynch JA, Buckland-Wright JC, Macfarlane DG (1993). Precision of joint space width measurement in knee osteoarthritis from digital image analysis of high definition macroradiographs. Osteoarthritis Cart.

[B23] Buckland-Wright JC, Bird CF, Ritter-Hrncirik CA, Cline GA, Tonkin C, Hangartner TN, Ward RJ, Meyer JM, Meredith MP (2003). X-ray technologists' reproducibility from automated measurements of the medial tibiofemoral joint space width in knee osteoarthritis for a multicenter, multinational clinical trial. J Rheumatol.

[B24] Cicuttini FM, Wluka AE, Stuckey SL (2001). Tibial and femoral cartilage changes in knee osteoarthritis. Ann Rheum Dis.

[B25] Beltran J (1990). The Knee. MRI of the Musculoskeletal System.

[B26] Resnick D, Kang HS, Catherine Fix (1997). Chapter 16, The knee. Internal Derangements of Joints: Emphasis on MR imaging.

[B27] Garnero P, Ayral X, Rousseau JC, Christgau S, Sandell LJ, Dougados M, Delmas PD (2002). Uncoupling of type II collagen synthesis and degradation predicts progression of joint damage in patients with knee osteoarthritis. Arthritis Rheum.

[B28] Christensen R, Astrup A, Bliddal H (2005). Weight loss: the treatment of choice for knee osteoarthritis? A randomized trial. Osteoarthritis Cartilage.

[B29] Pavelka K, Gatterova J, Altman RD (2000). Radiographic progression of knee osteoarthritis in a Czech cohort. Clin Exp Rheumatol.

[B30] Cicuttini FM, Forbes A, Yuanyuan W, Rush G, Stuckey SL (2002). Rate of knee cartilage loss after partial meniscectomy. J Rheumatol.

[B31] Felson DT, Lawrence RC, Dieppe PA, Hirsch R, Helmick CG, Jordan JM, Kington RS, Lane NE, Nevitt MC, Zhang Y (2000). Osteoarthritis: new insights. Part 1: the disease and its risk factors. Ann Intern Med.

[B32] Duryea J, Zaim S, Genant HK (2003). New radiographic-based surrogate outcome measures for osteoarthritis of the knee. Osteoarthritis Cartilage.

[B33] Vignon E, Piperno M, Le Graverand MP, Mazzuca SA, Brandt KD, Mathieu P, Favret H, Vignon M, Merle-Vincent F, Conrozier T (2003). Measurement of radiographic joint space width in the tibiofemoral compartment of the osteoarthritic knee: comparison of standing anteroposterior and Lyon schuss views. Arthritis Rheum.

[B34] Bruyere O, Honore A, Ethgen O, Rovati LC, Giacovelli G, Henrotin YE, Seidel L, Reginster JY (2003). Correlation between radiographic severity of knee osteoarthritis and future disease progression. Results from a 3-year prospective, placebo-controlled study evaluating the effect of glucosamine sulfate. Osteoarthritis Cartilage.

[B35] Hunter DJ, March L, Sambrook PN (2003). The association of cartilage volume with knee pain. Osteoarthritis Cartilage.

[B36] Wluka AE, Wolfe R, Stuckey S, Cicuttini FM (2004). How does tibial cartilage volume relate to symptoms in subjects with knee osteoarthritis?. Ann Rheum Dis.

[B37] Boegard T, Rudling O, Petersson IF, Jonsson K (1998). Correlation between radiographically diagnosed osteophytes and magnetic resonance detected cartilage defects in the tibiofemoral joint. Ann Rheum Dis.

[B38] Bhattacharyya T, Gale D, Dewire P, Totterman S, Gale ME, McLaughlin S, Einhorn TA, Felson DT (2003). The clinical importance of meniscal tears demonstrated by magnetic resonance imaging in osteoarthritis of the knee. J Bone Joint Surg Am.

[B39] Link TM, Steinbach LS, Ghosh S, Ries M, Lu Y, Lane N, Majumdar S (2003). Osteoarthritis: MR imaging findings in different stages of disease and correlation with clinical findings. Radiology.

[B40] Biswal S, Hastie T, Andriacchi TP, Bergman GA, Dillingham MF, Lang P (2002). Risk factors for progressive cartilage loss in the knee: a longitudinal magnetic resonance imaging study in forty-three patients. Arthritis Rheum.

[B41] Felson DT, Chaisson CE, Hill CL, Totterman SM, Gale ME, Skinner KM, Kazis L, Gale DR (2001). The association of bone marrow lesions with pain in knee osteoarthritis. Ann Intern Med.

[B42] Garnero P, Piperno M, Gineyts E, Christgau S, Delmas PD, Vignon E (2001). Cross sectional evaluation of biochemical markers of bone, cartilage, and synovial tissue metabolism in patients with knee osteoarthritis: relations with disease activity and joint damage. Ann Rheum Dis.

[B43] Ding C, Garnero P, Cicuttini F, Scott F, Cooley H, Jones G (2005). Knee cartilage defects: association with early radiographic osteoarthritis, decreased cartilage volume, increased joint surface area and type II collagen breakdown. Osteoarthritis Cartilage.

